# A Sad Sight

**Published:** 1855-12

**Authors:** 


### A SAD SIGHT.

"It is one of the saddest in nature, to see the old spiteful and vindictive, querulous with all." Said a sunny-faced little girl tc her mother one day:

"Mother, will grandfather go to heaven when he dies?"

"Yes, my child, I hope he will."

"Well, then I don't want to go there, for he is so cross."

Surely, time should mellow the heart of the aged, should fill it with sympathies, making it beam with forbearance towards all; and thus it is with the guileless and the good. Those who are not so, we should pity; for we may be sure they have missed the aim of life, have sown the seeds of wrong-doing in youth, which now, in their age, are bearing the wormwood and gall of the memory of ill-directed energies, of unjust purposes, of unholy ambitions, and it may be, of loving hearts, long ago wounded or broken by confidences falsified. Pity then, the malignant old man, who deals in the vile innuendo, drives a dagger from behind, or prostitutes a chance power, to feed his own hate; to such we say, be silent, pity and forgive.

